# miR-K12-7-5p Encoded by Kaposi's Sarcoma-Associated Herpesvirus Stabilizes the Latent State by Targeting Viral ORF50/RTA

**DOI:** 10.1371/journal.pone.0016224

**Published:** 2011-01-20

**Authors:** Xianzhi Lin, Deguang Liang, Zhiheng He, Qiang Deng, Erle S. Robertson, Ke Lan

**Affiliations:** 1 Key Laboratory of Molecular Virology and Immunology, Institut Pasteur of Shanghai, Shanghai Institutes for Biological Sciences, Chinese Academy of Sciences, Shanghai, People's Republic of China; 2 Department of Microbiology and the Abramson Comprehensive Cancer Center, University of Pennsylvania Medical School, Philadelphia, Pennsylvania, United States of America; Hannover Medical School, Germany

## Abstract

Seventeen miRNAs encoded by Kaposi's sarcoma-associated herpesvirus (KSHV) have been identified and their functions have begun to be characterized. Among these miRNAs, we report here that miR-K12-7 directly targets the replication and transcription activator (RTA) encoded by open reading frame 50. We found that miR-K12-7 targeted the RTA 3′ untranslated region (RTA3′UTR) in a seed sequence-dependent manner. miR-K12-7-5p derived from miR-K12-7 mediates the inhibition of RTA expression, and the mutation of the seed match site totally abrogated the inhibitory effect of miR-K12-7 on RTA3′UTR. The inhibition of RTA expression by miR-K12-7 was further confirmed in the latently KSHV-infected 293/Bac36 cell line through transient transfection of miR-K12-7 expression plasmid or specific inhibitor of miR-K12-7-5p, respectively. The transient transfection of miR-K12-7 into 293/Bac36 cells reduced RTA expression and the expression of the downstream early genes regulated by RTA, and also the production of progeny virus was significantly reduced after treatment with chemical inducers. Our study revealed that another miRNA, miR-K12-7-5p, targets the viral immediate early gene *RTA* and that this miRNA contributes to the maintenance of viral latency.

## Introduction

miRNAs are a class of small (∼21–25 nucleotides) single-stranded noncoding RNAs that posttranscriptionally regulate the expression of specific messenger RNAs by pairing to complementary target sequences within related mRNAs [1]. To date, more than 15,000 mature miRNAs have been discovered in 142 species (http://www.mirbase.org/, Release 16, Sep. 2010), which are involved in development, apoptosis, metabolism, embryonic patterning, and miRNA biogenesis [2]. Many viruses, like their hosts, encode miRNAs, which can be utilized by viruses to inhibit host cell apoptosis, evade the host immune system, regulate the viral life cycle, and control viral gene expression [3].

Kaposi's sarcoma-associated herpesvirus (KSHV) has been reported to express 17 mature miRNAs from 12 pre-miRNAs during viral latency [4], [5], [6], [7], but the functions of these are as yet largely unknown. Because these miRNAs were identified as components of the viral latency program, we speculate that they may contribute to the maintenance of viral latency. Therefore, we screened these miRNAs using a luciferase reporter construct containing the 3′ untranslated region (3′UTR) of the *RTA* gene (RTA3′UTR). Among these miRNAs, we found that miR-K12-7, together with miR-K12-9, regulates the switch between viral latency and lytic replication by directly inhibiting the expression of RTA, the major regulator of the viral life cycle. The regulation of RTA by miR-K12-7 and other miRNAs constitutes another mechanism by which KSHV maintains a latent state in host cells.

## Results

### miR-K12-7 targets the RTA3′UTR in reporter system

**Figure 1:**
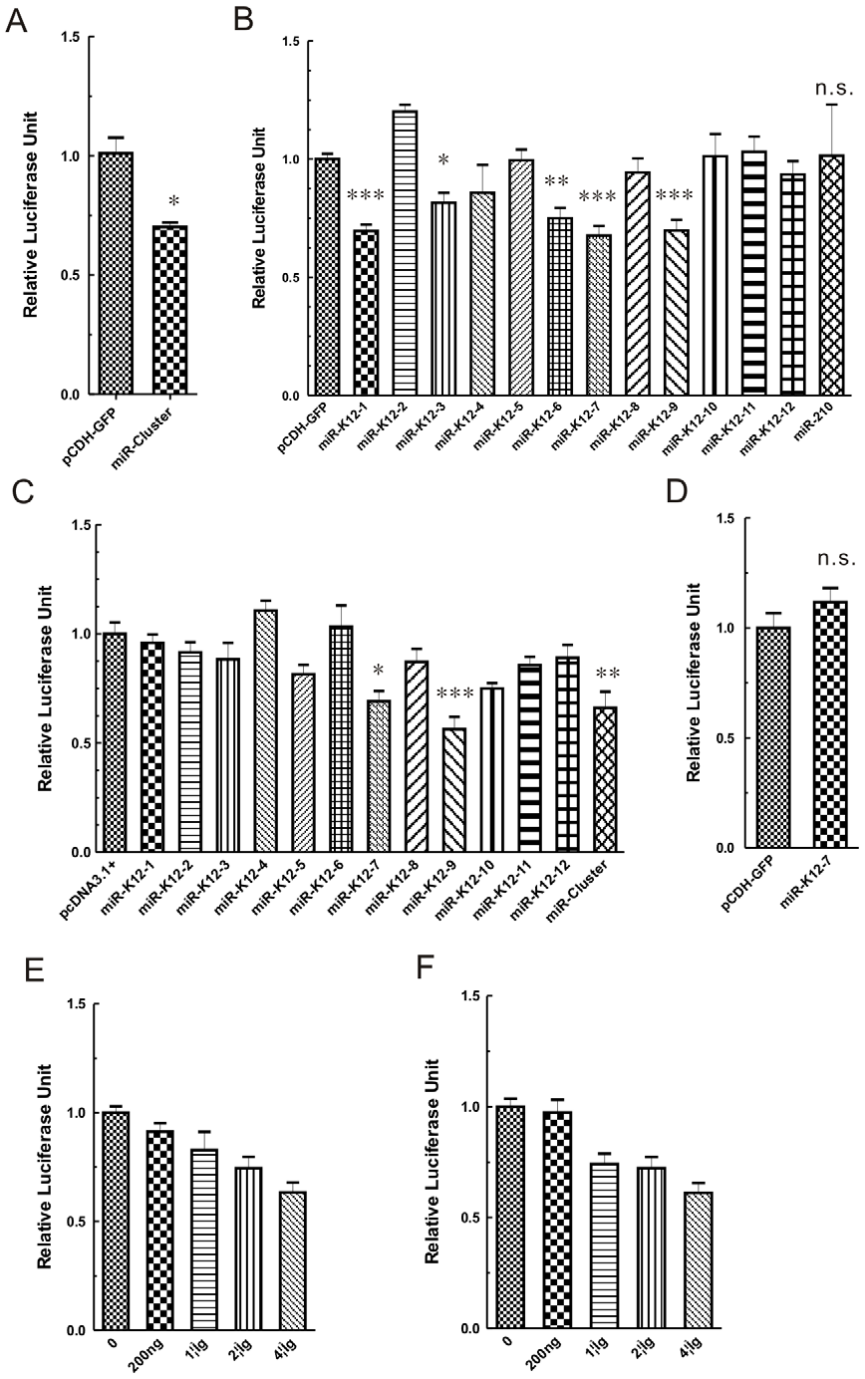
miR-K12-7 targets the RTA3′UTR. (A) miR-Cluster downregulates the expression of a reporter construct containing RTA3′UTR in HEK293 cells. HEK293 cells were cotransfected with pGL3–RTA3′UTR (50 ng) and miR-Cluster expression construct (1 µg) or the corresponding empty vector. The *Renilla* luciferase expression plasmid pRL–SV40 (4 ng) was transfected as the internal control. (B and C) miR-K12-7 and miR-K12-9 inhibited expression of a luciferase gene that was attached to RTA3′UTR in HEK293 cells or DG75 B-lymphoma cells, respectively. HEK293 (B) or DG75 (C) cells were cotransfected/coelectrotransfected with 50 ng/1 µg of pGL3–RTA3′UTR and 1 µg/20 µg of irrelevant hsa-miR-210 or individual miRNA expression construct or the corresponding empty vector. 4 ng/100 ng of pRL–SV40 was transfected as the internal control, respectively. D) miR-K12-7 does not target HDAC4-3′UTR. HEK293 cells were cotransfected with pGL3–HDAC4-3′UTR (400 ng) and miR-K12-7 expression construct (4 µg) or the corresponding empty vector. The *Renilla* luciferase expression plasmid pRL–SV40 (20 ng), was transfected as the internal control. E) and F) miR-Cluster (E) and miR-K12-7 (F) repressed the expression of the luciferase gene containing RTA3′UTR in a dose-dependent manner. HEK293 cells were cotransfected with 400 ng of pGL3–RTA3′UTR and increasing amounts (200 ng, 1, 2, or 4 µg) of the miR-Cluster or miR-K12-7 expression construct. Firefly and *Renilla* luciferase expression was measured 24 h after transfection. Firefly luciferase units were normalized to those of *Renilla* luciferase. Data are the mean ± SEM of six independent transfections. * *P*<0.05; ** *P*<0.01;*** *P*<0.001; n.s., not significant, *t-*test.

To test the hypothesis that the miRNAs expressed during the latent phase participate in the maintenance of viral latency, we decided to investigate the effects of these miRNAs on expression of RTA, the major regulator of the viral life cycle. We first amplified the RTA3′UTR [8] from the BCBL-1 cell line (primers are shown in Materials and Methods). RTA3′UTR was then inserted into the pGL3 vector downstream from the luciferase gene, and we designated this construct pGL3–RTA3′UTR. Each miRNA encoded by KSHV or a cluster of miRNAs (miR-Cluster, containing miR-K12-1 to miR-K12-9 and miR-K12-11) was cloned into the expression vector pcDNA3.1+ or pCDH-copGFP (System Biosciences, Mountain View, CA), as described previously [9] (Table 1). Above all, we tested the overall effects of miR-Cluster on RTA3′UTR. pCDH–GFP-miR-Cluster or the corresponding empty vector was cotransfected with pGL3–RTA3′UTR into HEK293 cells. The Renilla luciferase expression plasmid pRL–SV40 (Promega, Madison, WI) was transfected as an internal control. We found that the miR-Cluster expression plasmid reduced the expression of firefly luciferase from pGL3–RTA3′UTR by about 30% compared with the reduction caused by the empty vector pCDH-copGFP (Figure 1A). This suggests that these miRNAs, which were expressed in the cluster during viral latency, have an overall negative role in the regulation of RTA expression.

**Table 1:** Primer pairs for cloning KSHV pre-miRNA expression constructs.

Construct	Primer sequence		Genome position*
miR-K12-1	5′ primer	AATCTGGTTGACGGACTTTC	121783–121802
	3′ primer	GTACGCGGTTGTTTACGCAG	121947–121966
miR-K12-2	5′ primer	AGGCATTGTAGCTGTTGCGTT	121620–121640
	3′ primer	CGCTGCCACCTGCGTGTTCC	121809–121828
miR-K12-3	5′ primer	GTTCGTCGCTTGGACCTGGAG	121522–121542
	3′ primer	GTCCCCAAACTCCCAACCAA	121639–121658
miR-K12-4	5′ primer	GAGGTTTGAGAGGCGTAGACATCC	121364–121387
	3′ primer	CTCCAGGTCCAAGCGACGAAC	121522–121542
miR-K12-5	5′ primer	CCCGCATAGGTTTTTGTGG	121216–121234
	3′ primer	GGATGTCTACGCCTCTCAAACCTC	121364–121387
miR-K12-6	5′ primer	ACACAGAACAATAACGGGCGACTA	120727–120750
	3′ primer	TAAAGCGGGCGTTCGTAAGC	120861–120880
miR-K12-7	5′ primer	CTACACTAAGCCCGAACG	120293–120310
	3′ primer	CGTGCCCACCGATGAGATAC	120446–120465
miR-K12-8	5′ primer	TAGCAGGGCCATCCACAC	119884–119901
	3′ primer	TGACAAAGCATGCACTGGAAATC	120031–120053
miR-K12-9	5′ primer	TGCTTCCGGAAATACCACCTGAGT	119220–119243
	3′ primer	TGAGTCATCGCAGCCCCTATTC	119384–119405
miR-K12-10	5′ primer	GCAACTCGTGTCCTGAATGCTA	117935–117956
	3′ primer	CTCCTCACTCCAATCCCAAT	118104–118123
miR-K12-11	5′ primer	AAAAATTGCCGCCGTGAAGGTC	120520–120541
	3′ primer	TTCATCATTTCACCCACCGTCTCT	120699–120722
miR-K12-12	5′ primer	TTGGGAGAGCGGACGCCA	117606–117623
	3′ primer	CATACAATGCTGAAGAGCAGG	117816–117836
miR-Cluster	5′ primer	TCCCAGTAGAGTGACCCAG	119099–119117
	3′ primer	GTACGCGGTTGTTTACGCAG	121947–121966

* All positions indicated here were referenced in the KSHV genome (accession number: U75698).

To identify which miRNAs are responsible for downregulation of RTA3′UTR expression, each miRNA expression construct or the corresponding empty vector, or an irrelevant human miRNA hsa-miR-210 was cotransfected with pGL3–RTA3′UTR into HEK293 cells. Several miRNAs, including miR-K12-1, miR-K12-3, miR-K12-6, miR-K12-7 and miR-K12-9, repressed to varying degrees the expression of the reporter gene that contained RTA3′UTR (Figure 1B). miR-K12-7 reduced expression of the reporter most effectively in our experimental system (Figure 1B). We repeated this experiment in the DG75 B-lymphoma cell line instead of HEK293 cells. A similar repressive effect on reporter gene expression was observed for miR-K12-7 and miR-K12-9, but not for miR-K12-1, miR-K12-3 and miR-K12-6 (Figure 1C), which suggests that the effect of miR-K12-7 and miR-K12-9 was not cell-type restricted. It has already been reported that miR-K12-9 is able to downregulate RTA expression directly [10], therefore, we focused on miR-K12-7. The repressive effect of miR-K12-7 on expression of the reporter gene that contained RTA3′UTR was specific because miR-K12-7 only repressed expression of the reporter gene with RTA3′UTR, but not that with HDAC4-3′UTR, which lacked the target site in its sequence (Figure 1D). Downregulation of RTA3′UTR by miR-K12-7 or miR-Cluster was also miRNA-dose dependent (Figure 1E and 1F), which indicates that RTA3′UTR is targeted by miR-Cluster and miR-K12-7.

### The seed-match site is essential for the regulation of RTA by miR-K12-7-5p

**Figure 2:**
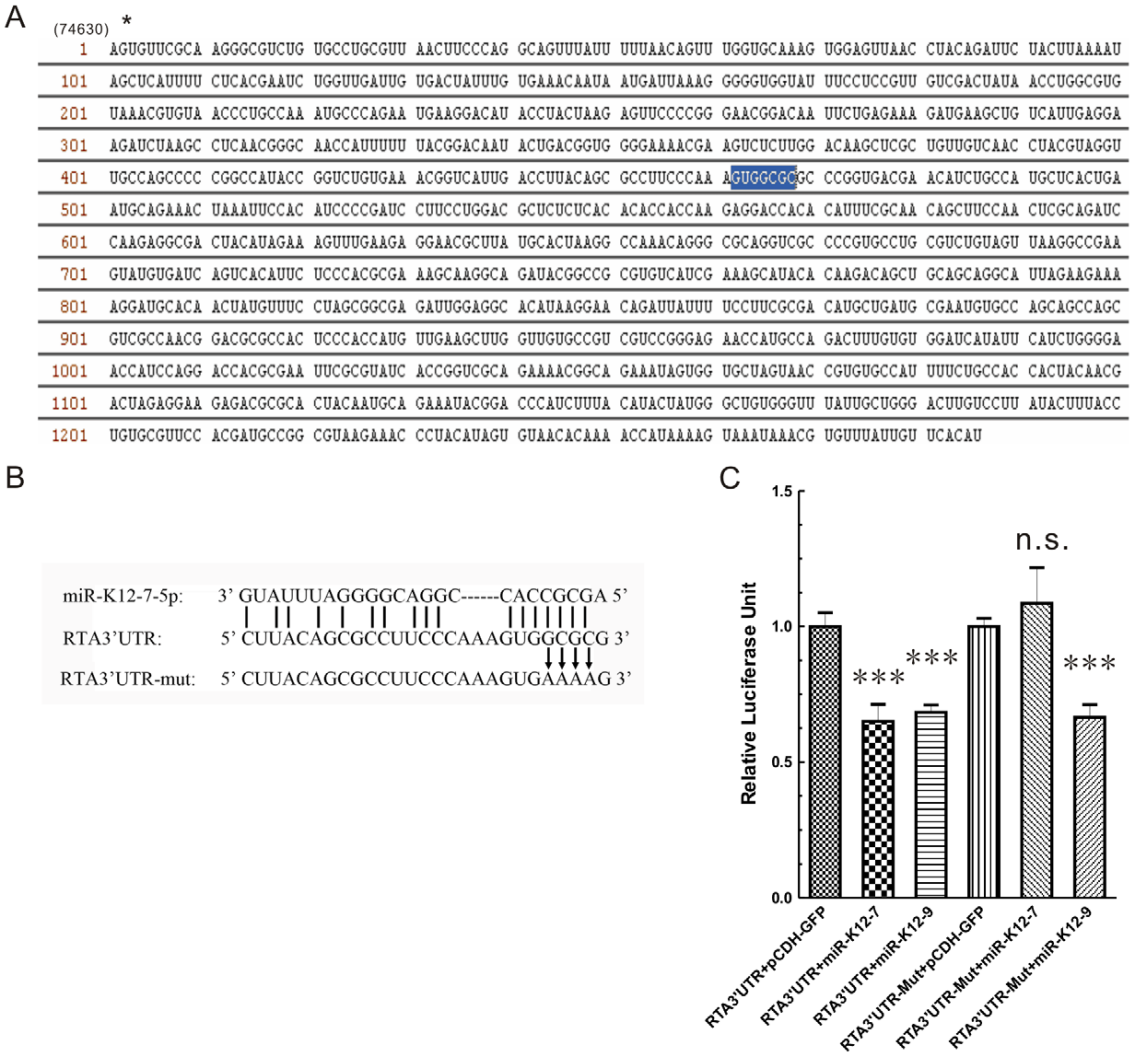
The seed-match site is essential for the regulation of RTA by miR-K12-7-5p. (A) The type I RTA 3′UTR (nt 1–1286) contains one perfect seed-match site (highlighted). *74630 indicate the starting site position in the KSHV genome (accession number: U75698). (B) Potential base pairing between miR-K12-7-5p and its predicted pairing site in RTA3′UTR. Watson–Crick base pairs are indicated with vertical lines. The mutations made in the 3′UTR are shown below the wild-type sequences. (C) Site-directed mutagenesis demonstrates that the seed match site is essential for the regulation of RTA expression by miR-K12-7-5p. HEK293 cells were cotransfected with 50 ng pGL3–RTA3′UTR or pGL3–RTA3′UTR-mut and 1 µg miR-K12-7 or miR-K12-9 expression construct, or the corresponding empty vector. pRL–SV40 (4 ng) was transfected as the internal control. Luciferase activity was measured 24 h after transfection. The data shown are from six independent transfections. ***P<0.001; n.s., not significant, *t-*test.

Because the seed sequence (nucleotides [nt] 2–7/8) is critical for miRNA function [11], the seed complementarity for miR-K12-7 at the 3′UTR of the RTA mRNA was determined. Initially, miR-K12-7 is considered to encode only one mature miRNA, designated “miR-K12-7-3p” [4], [5], [6]. More recently, using deep sequencing technology, two groups separately found that miR-K12-7 can actually gives rise to two mature miRNAs, miR-K12-7-5p and miR-K12-7-3p, in different KSHV-positive cell lines [12], [13]. We confirmed this result with bulge–loop quantitative PCR (qPCR) by checking the total RNA samples from KSHV-positive cell lines BCBL-1, BC-3 and JSC-1, or the total RNA from HEK293 cells transfected with an miR-K12-7 expression construct, respectively (Figure S2; the bulge–loop qPCR primers were synthesized by RiboBio, Inc., Guangzhou, China). When we searched the 3′UTR of RTA mRNA for seed complementarity to miR-K12-7, we identified a 7-mer complementary to nt 2–8 of miR-K12-7-5p (Figure 2A).

Since other group had already shown that miR-K12-7-3p didn't target RTA3′UTR by using either miR-K12-7-3p mimics or inhibitor [10], we supposed that the inhibitory effect of miR-K12-7 expression plasmid on the expression of luciferase gene bearing RTA3′UTR was possibly mediated by miR-K12-7-5p. Mutational analysis confirmed our hypothesis that the observed inhibition of luciferase expression by miR-K12-7 is dependent on the presence of the seed-match site in RTA3′UTR. Mutations were introduced at nt 2–5 of the 3′UTR of RTA mRNA to reduce its Watson–Crick base pairing with miR-K12-7-5p (Figure 2B). HEK293 cells were cotransfected with pGL3–RTA3′UTR or pGL3–RTA3′UTR-mut and miR-K12-7 or miR-K12-9 expression plasmid, or the corresponding empty vector. The Renilla expression plasmid pRL–SV40 was also transfected as an internal control. Firefly luciferase activity was measured and normalized to the amount of Renilla luciferase activity. When HEK293 cells were cotransfected with the wild-type RTA3′UTR, miR-K12-7 and miR-K12-9 significantly reduced luciferase expression (Figure 2C), similar as results shown in Figure 1B and 1C. However, when pGL3–RTA3′UTR-mut and the miR-K12-7 or miR-K12-9 expression plasmid were cotransfected into HEK293 cells, the mutation of the seed-match site totally abrogated the suppressive effect of miR-K12-7 on the expression of the luciferase gene, whereas the effect of miR-K12-9 was not affected (Figure 2C). This is probably attributable to the destruction of the potential pairing between miR-K12-7-5p and the predicted pairing site in RTA3′UTR (Figure 2B). The result also suggests that miR-K12-9 probably targets different sites from those targeted by miR-K12-7 in RTA3′UTR. These data indicate that the seed-match site is critical for the targeting of miR-K12-7-5p to RTA3′UTR.

### miR-K12-7 reduces RTA expression in KSHV-positive 293/Bac36 cells

**Figure 3:**
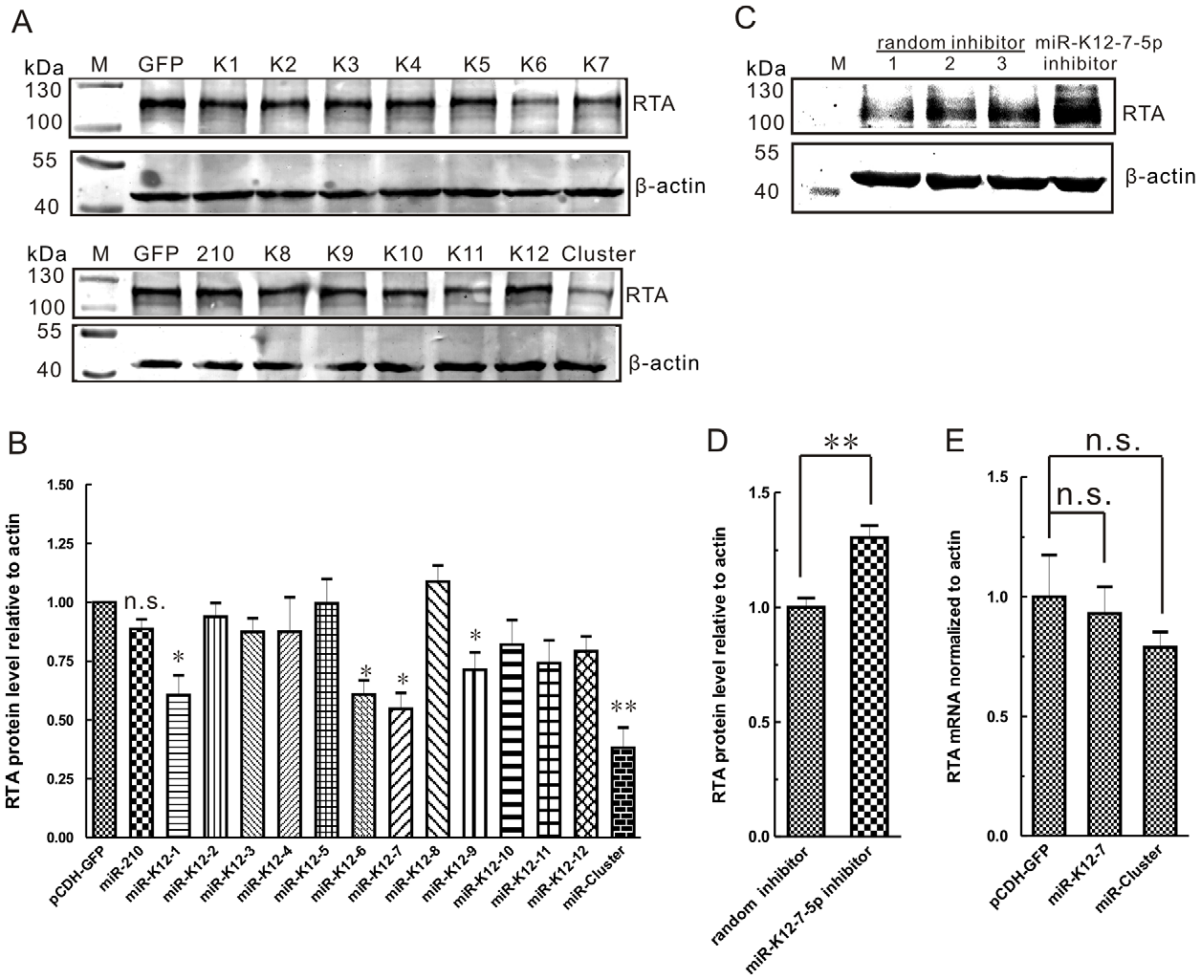
miR-K12-7 inhibits RTA expression at the protein level but not at the mRNA level. (A) The transient expression of miR-K12-7 repressed RTA protein expression in the latently KSHV-infected 293/Bac36 cell line. Latently KSHV-infected 293/Bac36 cells were synchronized by culture in serum-free medium and pretransfected with 4 µg of individual miRNA-expressing constructs or the corresponding empty vector 12 h after the addition of serum. After a further 4 h, the S-phase 293/Bac36 cells were induced to lytic replication with both TPA (25 ng/mL) and VPA (3 mM). Cell lysates were prepared 26 h after induction and were probed for endogenous RTA or β-actin. (B) A graph of RTA levels normalized to those of β-actin of three independent experiments performed in Figure 3A (analyzed with NIH ImageJ). (C) miR-K12-7-5p inhibitor increases RTA expression. Synchronized 293/Bac36 cells were transfected with 200 nM miR-K12-7-5 inhibitor or random inhibitor and were introduced into lytic replication with both TPA (25 ng/mL) and VPA (3 mM). Cell lysates were prepared 48 h after induction and were probed for endogenous RTA or β-actin. (D) A graph of RTA levels normalized to those of β-actin of three independent experiments performed in Figure 3C (analyzed with NIH ImageJ). (E) RTA mRNA levels were not affected by miR-K12-7. At 26 h after induction with TPA and VPA, the 293/Bac36 cells transfected with individual miRNA expression constructs or the corresponding empty vector were harvested and RNAs were extracted from those cells with TRIzol Reagent. RTA mRNA from those cells was assayed by RT–PCR and quantified relative to β-actin mRNA levels. The data shown are from triplicate independent experiments. *P<0.05; ** *P*<0.01; n.s., not significant. *t* test.

Because the experiments described above showed that miR-K12-7 is a negative regulator of RTA3′UTR, we next examined whether miR-K12-7 is an authentic miRNA with inhibitory effect on RTA expression. The 293/Bac36 cell, latently infected with KSHV, was transfected with individual miRNA expression construct or the corresponding empty vector or an irrelevant miRNA hsa-miR-210, and revealed that several miRNAs encoded by KSHV, miR-K12-1, miR-K12-6, miR-K12-7 and miR-K12-9 were all able to inhibit the expression of RTA to varying degrees (Figure 3A). Similar inhibition of RTA expression by miR-Cluster was also observed (Figure 3A). Quantification of these effects from three independent experiments (Figure S3) indicated that among these miRNAs, miR-K12-7 repressed RTA expression most significantly, by >45% (Figure 3B; analyzed with NIH ImageJ). miR-Cluster reduced the expression of RTA by >60% (Figure 3B), which was stronger than with any of the individual miRNAs, and was probably due to a combined effect of these miRNAs. To confirm that miR-K12-7-5p is a negative regulator of *RTA*, we then transfected 293/Bac36 cells with inhibitor specific to miR-K12-7-5p or random inhibitor (purchased from RiboBio, Inc., Guangzhou, China), respectively. miR-K12-7-5p inhibitor increased RTA expression significantly compared with random inhibitor (Figure 3C) and showed about 30% increase of RTA expression (Figure 3D; analyzed with NIH ImageJ). These results demonstrate that miR-K12-7-5p can inhibit expression of RTA in a KSHV latently infected cell line.

### miR-K12-7 inhibits RTA expression at the protein level but not at the mRNA level

Because miRNA can repress its target at both the transcriptional and translational levels, we next examined whether KSHV-encoded miRNAs target RTA at the RNA level. Total RNAs were harvested at the same timepoint at which Protein samples were collected from 293/Bac36 cells transfected with the individual miRNA expression constructs or the corresponding empty vector. Primers for RTA and β-actin cDNA were used to evaluate the RTA and β-actin mRNA levels with real-time PCR. The RTA mRNA level was then normalized to the β-actin mRNA level, and the relative quantity of RTA mRNA in 293/Bac36 cells transfected with pCDH-GFP was used as the control value. If the complementarity between the miRNA and the target 3′UTR is low, the miRNA will usually inhibit the expression of the target gene translationally [11]. We found that miR-K12-7 did not affect the levels of RTA mRNA (Figure 3E), which suggests that miR-K12-7 regulates the expression of RTA at the translational level.

### miR-K12-7 downregulates RTA downstream genes expression and impairs the production of progeny virus

**Figure 4:**
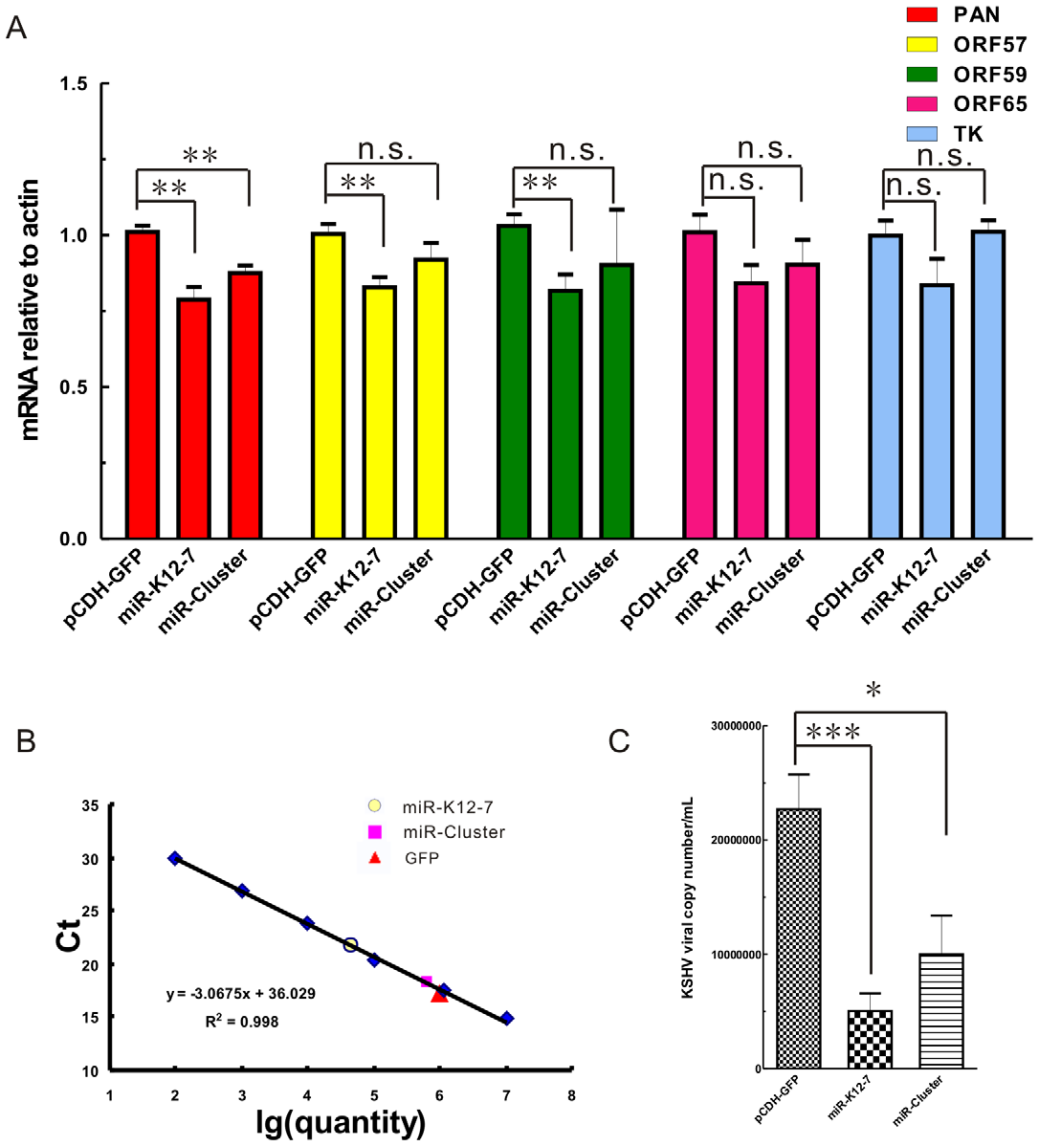
miR-K12-7 downregulates the expression of the genes downstream from RTA and impairs the production of progeny virus. (A) RNA levels of the early genes *PAN*, *ORF57*, and *ORF59* were slightly downregulated by miR-K12-7 and those of the late genes *ORF65* and *TK* were unaffected by miR-K12-7. Forty-eight hours after induction with TPA and VPA, the 293/Bac36 cells transfected with the individual miRNA expression constructs or the corresponding empty vector were harvested and total RNA was extracted from them with TRIzol Reagent. RNA levels of the early genes *PAN*, *ORF57*, and *ORF59* and the late genes *ORF65* and *TK* were assayed by RT–PCR and quantified relative to β-actin mRNA levels. The data shown are from triplicate independent experiments. (B) The standard curve for virion quantification used to calculate the number of KSHV virions in the supernatants of miRNA-transfected 293/Bac36 cells. (C) miR-K12-7 significantly impaired the production of progeny virus in the supernatant of 293/Bac36 cells after chemical induction. Latently KSHV-infected 293/Bac36 cells synchronized by culture in serum-free medium were pretransfected with 4 µg of individual miRNA-expressing constructs or empty vector 12 h after the addition of serum. After a further 4 h, the S-phase 293/Bac36 cells were induced to lytic replication with both TPA (25 ng/mL) and VPA (3 mM). The viral genomes from the supernatants were extracted five days after induction and were assayed by real-time PCR. The viral copy numbers were calculated from the standard curve derived from the ORFK9 construct. The data shown are from triplicate independent experiments. *P<0.05; **P<0.01; *** *P*<0.001; n.s., not significant. *t* test.

The experiments described above show that miR-K12-7 negatively modulates RTA3′UTR, and imply that miR-K12-7 has some functional role in the regulation of the KSHV life cycle. As we already know, the immediate early gene product RTA is a master molecule that responds quickly to stimuli [8]. RTA activates downstream genes, including PAN RNA, kaposin (K12), open reading frame 57 (ORF57), ORF59, thymidine kinase, vIL-6, etc., in a cascade fashion [14], [15], [16], [17], [18], [19]. We then examined the effect of miR-K12-7 on the expression of genes downstream from RTA using quantitative real-time PCR. We found at 48 h that the expression levels of those early downstream genes regulated by RTA were slightly reduced by miR-K12-7 upon chemical induction with 12-O-tetradecanoylphorbol-13-acetate (TPA) and valproic acid (VPA), whereas the expression of the late genes downstream from RTA were not affected (Figure 4A). This suggests that miR-K12-7 mildly affects the expressions of the downstream genes regulated by RTA by targeting RTA.

We next investigated the effects of miR-K12-7 on the production of progeny virus after chemical induction. On day 5 after induction with TPA and VPA, the supernatants of 293/Bac36 cells, pretransfected with the miR-K12-7 expression construct or the corresponding empty vector, were collected. The viral genomes were then extracted from the supernatants and assayed by real-time PCR. The viral copy numbers were calculated according to the standard curve derived from the ORFK9 construct (Figure 4B). We found that miR-K12-7 reduced the production of progeny virus from 293/Bac36 cells by more than four fold compared with that of empty vector (Figure 4C). Interestingly, miR-Cluster also showed more than two fold reduction of progeny virus (Figure 4C). Taken together, these data suggest that miR-K12-7 prevents the production of progeny virus by targeting RTA, which may help to maintain a latent state of viral infection.

## Discussion

Since the discovery of KSHV, the regulation of its latency–lytic replication status has intrigued researchers in the field. During latent infection, the viral episome is tethered to the host chromosomes and replicates with the host cell. At this stage, very few viral genes are expressed. Those that are expressed include the genes encoding viral FLIP (*vFLIP*), viral cyclin (*vCYC*), and latency-associated nuclear antigen (*LANA*; see review [20]). LANA has previously been reported to inhibit lytic replication by targeting the immediate early gene *RTA*, in a mechanism that controls virus-mediated latency [21].

Recently, studies in several laboratories have shown that besides those typical latency genes, KSHV also encodes 12 pre-miRNAs, which can give rise to 17 mature miRNAs, during latency [4], [5], [6], [7]. Early observations have suggested a role for miRNAs in the regulation of the latency-to-lytic-replication switch in herpes simplex virus 1 (HSV1) [22]; and human cytomegalovirus (HCMV) [23]. One of those studies demonstrated that at least two miRNAs encoded by HSV1, miR-H2-3p and miR-H6, which are expressed in latently infected neurons, facilitate the establishment and maintenance of viral latency by posttranscriptionally regulating the expression of two viral immediate early genes, *ICP0* and *ICP4.5*, respectively [22]. Another study demonstrated that miR-UL112-1, encoded by HCMV, contributes to the control of viral latency by regulating the viral immediate early gene *IE1* [23]. Based on the strong similarities among herpesviruses, we speculated that miRNAs encoded by KSHV may also play a similar role. Because *RTA* is the only gene essential for the switch from latency to lytic replication (see review [24]), we hypothesized that miRNAs encoded by KSHV target this immediate early gene.

In this study, we found that the miRNA encoded by KSHV, miR-K12-7-5p, newly identified by deep sequencing [12], [13] and confirmed by bulge–loop quantitative PCR in our laboratory (Figure S1 and S2), targets expression of RTA at translational level. This inhibitory function of miR-K12-7-5p on the expression of RTA is dependent upon the miRNA seed sequence (Figure 2C). We also demonstrated that miR-K12-7-5p slightly reduces the expression of the downstream early genes regulated by RTA (*PAN*, *ORF57*, and *ORF59*) at 48 h post chemical induction (Figure 4A) and significantly reduces the production of progeny virus in the 293/Bac36 cell line (Figure 4C), which is latently infected with KSHV, upon chemical induction. We also confirmed that miR-K12-9 repressed the expression of RTA; both in the reporter system and in KSHV latently infected 293/Bac36 cells. The target site of miR-K12-9 in RTA3′UTR differs from that of miR-K12-7-5p. We also observed that miR-K12-1 and miR-K12-6 repressed the expression of RTA in KSHV latently infected 293/Bac36 cells (Figure 3A) but not in one of our reporter systems (Figure 1C), which suggests that these two miRNAs affect RTA indirectly, as reported previously [25]. Taken together, these data demonstrate that miR-K12-7-5p, encoded by KSHV, can facilitate the maintenance of the latent viral state by translationally regulating the expression of the viral immediate early gene *RTA*.

While this manuscript was in preparation, several other groups reported some other miRNAs encoded by KSHV also target *RTA*. Briefly, Bellare et al. reported that RTA expression is inhibited by miR-K12-9*, which directly targets the 3′UTR of RTA mRNA [10]. Two other groups reported that the expression of RTA can be indirectly regulated by both miR-K12-5 [26] and miR-K12-1 [25], although the underlying mechanisms are totally different. In our study, we confirmed that miR-K12-9 was able to repress expression of the reporter gene that contained RTA3′UTR in two different cell lines (Figure 1B and 1C), and miR-K12-9 was also able to repress expression of RTA in KSHV latently infected 293/Bac36 cells (Figure 3A). We did not subsequently study the effect of miR-K12-1 and miR-K12-6 because we thought that both of these miRNAs would target RTA indirectly because they did not show similar inhibition of expression of the reporter gene in DG75 cells (Figure 1C). However, we noticed that miR-K12-1 and miR-K12-6 both repressed RTA expression in both HEK293 cells (Figure 1B) and the KSHV latently infected cell line 293/Bac36 (Figure 3A).

It is interesting to notice that four groups discovered four different miRNAs which all were able to target RTA, directly or indirectly. By comparing the different experimental systems and conditions among us four groups, we think the different results are understandable. The previous analysis from Bellare *et al*. employed miRNA mimics rather than pre-miRNA expression plasmids used by other three groups [10]. Specifically, for miR-K12-7, Bellare *et al*. only used mimics or specific inhibitor for miR-K12-7-3p since miR-K12-7-5p had not yet been discovered at the time of their study [10]. Although Lu *et al*. used the same pre-miRNA expression plasmids as we did, they didn't carry out a comprehensive screen for the effect of individual miRNA encoded by KSHV on expression of reporter gene bearing RTA3′UTR. Moreover, Lu *et al*. focused more on identifying those miRNAs that were found to be able to inhibit RTA expression at the transcriptional level [26]. Lei *et al*. identified NF-κB inhibitor IκBα as a direct target of miR-K12-1 and expression of miR-K12-1 enhanced NF-κB activity [25]. Based on known evidences that NF-κB was a strong negative regulator of RTA transactivation through forming complex with RBP-Jκ (a major coactivator of RTA) [27], they proposed a working model that miR-K12-1 suppresses viral lytic replication via activation of NF-κB [25]. This model really makes sense but it doesn't exclude the possibility that *RTA* can be directly targeted by other miRNAs encoded by KSHV. And this model could not fully explain that why RTA was highly upregulated in ΔmiRs (Figure 1a and 1b in Ref. [25]) since NF-κB has no direct known effect on RTA expression. Although Lei *at al*.showed no effects of KSHV miRNAs on all three types of RTA3′UTR in their supporting information [25], we could not repeat their results due to lacking of dosage parameters for plasmids involved in. Taken together, based on different experimental systems, we and Bellare *et al*. have identified two different miRNAs that are encoded by KSHV, that are both able to inhibit RTA expression translationally, through direct targeting of RTA3′UTR. By using miR-Cluster deletion mutant, Lu *et al*. and Lei *et al*. have discovered two distinct miRNAs that are encoded by KSHV that can indirectly repress RTA expression through different mechanisms.

Many miRNAs that are encoded by KSHV contribute to regulation of the viral switch between latency–lytic replication by targeting the viral immediate early gene *RTA*, using different mechanisms simultaneously. A further question is why KSHV encodes so many miRNAs that are all able to downregulate expression of RTA, which is a major regulator of the viral life cycle. One reasonable explanation is that the virus requires backup systems to maintain viral latency safely. This makes sense because, as well as the role played by miRNAs in the regulation of the switch between latency and lytic replication, LANA is known to function in the maintenance of latency by repressing the promoter of *RTA*, and by binding directly to the RTA protein to prevent its self-reactivation [21]. Another potential explanation is that a single miRNA might not be strong enough to repress RTA expression to maintain viral latency, and combined effects are required. This is supported by the fact that the effects of all miRNAs that are reported to target RTA are marginal (Figure 3A) [10], [25], [26], and all miRNAs that target RTA expression, directly or indirectly, are expressed at relatively low levels compared with the most abundantly expressed miRNAs, miR-K12-4-3p in BC3 cells or miR-K12-6-3p in TRE-BCBL-1 cells [12], [13] (Figure S1). Further studies are required to elucidate the contributions of individual miRNAs that are encoded by KSHV to regulation of the viral life cycle. Single and/or combined miRNA mutation or deletion in the context of the whole viral genome might be the best way to understand the effect of each miRNA on RTA expression and viral latency maintenance.

We also noted that there are two different facets for the effects of miR-K12-5 on the latency-to-lytic replication switch: one promotes maintenance of latency in cells [26], and the other facilitates lytic reactivation in cells [28]. Further studies are needed to investigate which effect is dominant under different viral life cycle conditions. Studies from different groups that have reported several miRNAs encoded by KSHV that can repress expression of RTA by distinct mechanisms demonstrate the importance of RTA regulation, and add a new dimension to our understanding about the complex regulation networks for the KSHV viral life cycle. This complexity could provide a paradigm for the study of cellular and other viral miRNAs.

## Materials and Methods

### Cell culture and transfection

The human embryonic kidney (HEK) 293 cell line was grown in Dulbecco's modified Eagle's medium (DMEM) supplemented with 10% fetal bovine serum, 2 mM l-glutamine, 25 U/mL penicillin, and 25 µg/mL streptomycin. A HEK293 cell line that harbors the KSHV genome inserted into a bacterial artificial chromosome, designated 293/Bac36, was grown under the same conditions, except with the addition of 100 µg/mL hygromycin B. The cells were seeded the day before transfection to achieve ∼80%–90% confluence on the following day. DG75 (a KSHV-negative cell line) and BCBL-1 (a KSHV-positive cell line) were cultured in RPMI 1640 medium plus 10% fetal bovine serum. All the cells were cultured at 37°C in the presence of 5% CO_2_. HEK293 cells and 293/Bac36 cells were both transfected with Lipofectamine 2000 (Invitrogen, Carlsbad, CA), according to the manufacturer's protocol. DG75 cells were transfected by electroporation with a Bio-Rad Gene Pulser II electroporator (Bio-Rad Laboratories, Hercules, CA). A total of 5×10^6^ cells, harvested in exponential phase, were collected and washed with phosphate-buffered saline (PBS) and then resuspended in 400 µL of serum-free RPMI 1640 with the DNA mixture to be transfected. The resuspended cells were transferred to a 0.4 cm cuvette and electroporated at 975 µF and 220 V. The electroporated cells were transferred to 8 mL of complete medium, and then incubated at 37°C under 5% CO_2_.

### Constructs

Type I RTA3′UTR [8] was amplified by PCR from the total RNA extracted from the BCBL-1 cell line that had been induced with TPA for 36 h. Primers for RTA3′UTR were as follows: 5′ CCAggtaccAGTGTTCGCAAGGGCGTC; 3′ CCActcgagATGTGAACAATAAACACGTTTATT. The PCR products were excised with *Kpn*I and *Xho*I and cloned into pGL3cM. This plasmid was constructed by removing the multiple cloning site (MCS) from the pGL3 control vector with *Kpn*I and *Bgl*II, and infilling it with Klenow fragment, after which the in vitro MCS was inserted into the *Xba*I site. The new construct was designated pGL3–RTA3′UTR and confirmed by restriction enzyme digestion and DNA sequencing.HDAC4-3′UTR-pGL3 was o kind gift from Dr. Changsheng Du (Tongji University, China).

Stem–loop pre-miRNAs were PCR amplified from BCBL-1 genomic DNA, as described previously [9] (all primers for cloning KSHV pre-miRNAs as well as miR-Cluster are listed in Table 1). The PCR products were digested with *Xho*I and *Eco*RI or *Bam*HI and *Eco*RI, and inserted into pcDNA3.1+ (Invitrogen) or pCDH–copGFP (System Biosciences) at the corresponding sites. All these constructs were confirmed by both restriction enzyme digestion and DNA sequencing. Individual miRNA expression was confirmed by Bulge-Loop qPCR (Supplementary Figure S1).

### Dual-luciferase reporter assay

A Dual-Luciferase Reporter Assay System (Promega) was used to examine the effects of miRNAs on their target genes. Each miRNA-expressing plasmid or its corresponding vector (4 µg) was cotransfected with RTA3′UTR–pGL3cM (100 ng) and pRL–SV40 (2 ng; Promega) into HEK293 cells grown on a six-well plate using Lipofectamine 2000 transfection reagent (10 µL; Invitrogen). The transfected HEK293 cells were cultured for 24 h before harvesting for the luciferase assay. The cells were washed with PBS and homogenized with 200 µL of Passive Lysis Buffer (Promega, Madison, WI). Luciferase Assay Buffer (25 µL) was transferred into a 96-well plate. The lysate (10 µL) was added to two wells (duplicate) of the 96-well plate and mixed. Firefly luciferase activity was measured on a Veritas luminometer (Turner BioSystems, Sunnyvale, CA). The plate was removed from the luminometer and Stop & Glo Reagent (25 µL) was added and mixed. *Renilla* luciferase activity was measured on the luminometer. The data recorded on the luminometer were analyzed and graphed using Excel (Microsoft).

### Western blot analysis

Twenty-four hours after transfection, whole-cell lysates of 293/Bac36 cells were prepared with radioimmunoprecipitation assay (RIPA) buffer (50 mM Tris [pH 7.6], 150 mM NaCl, 2 mM EDTA, 1% Nonidet P-40, 1 mM phenylmethylsulfonyl fluoride, 1 µg/mL aprotinin, and 1 µg/mL pepstatin) for 1 h on ice, with brief vortexing every 15 min. The whole-cell lysates were then heated in sodium dodecyl sulfate (SDS)–mercaptoethanol lysis buffer and analyzed by SDS–polyacrylamide gel electrophoresis. The proteins were transferred from the gel to polyvinylidene difluoride membranes. The membranes were blocked with 5% dry nonfat milk for 1 h and probed with a mouse monoclonal antibody directed against RTA (using a monoclonal antibody raised by Yunhua Liu in our laboratory) at a 1∶1000 dilution, and an anti-β-actin antibody (I-19; Santa Cruz Biotechnology) at a 1∶1000 dilution. After the secondary antibody reaction, the membranes were washed in TBS–Tween (0.02 M Tris [pH 7.6], 0.1 M NaCl, 0.05% Tween-20) and visualized using a chemiluminescence reaction.

### RNA extraction and quantitative real-time PCR

The cells were harvested 24 h after transfection, and the total RNA was extracted with TRIzol Reagent (Invitrogen), according to the manufacturer's instructions. The cDNA (20 µL) for real-time PCR was generated from 4 µg of total RNA with the First-Strand cDNA Synthesis Kit (Fermentas UAB, Fermentas International Inc., Burlington, Canada), after priming with 1 µL oligo(dT)_18_, according to the manufacturer's instructions. The primer sequences for the real-time PCR system, which were designed and chosen with the Primer Express Software (version 3.0, Applied Biosystems, Inc., Foster City, CA), were as follows: RTA-RT-F, 5′-AGACCCGGCGTTTATTAGTACGT-3′; RTA-RT-R, 5′-CAGTAATCACGGCCCCTTGA-3′; ORF57-RT-F, 5′-TGGCGAGGTCAAGCTTAACTTC-3′; ORF57-RT-R, 5′-CCCCTGGCCTGTAGTATTCCA-3′; ORF59-RT-F, 5′-TTGGCACTCCAACGAAATATTAGAA-3′; ORF59-RT-R, 5′-CGGGAACCTTTTGCGAAGA-3′; PAN-RT-F, 5′-GCCGCTTCTGGTTTTCATTG-3′; PAN-RT-R, 5′-TTGCCAAAAGCGACGCA-3′; **ORF65-RT-F, 5′-GGCGGCCGTTTCCG-3′; ORF65-RT-R, 5′-TCATTGTCGCCGGCG-3′; β-actin-RT-F, 5′-TTGCCGACAGGATGCAGAAGGA-3′; β-actin-RT-R, 5′-AGGTGGACAGCGAGGCCAGGAT-3′.**

The real-time PCRs were performed with the SYBR Green Real-Time PCR Master Mix Kit (Toyobo, Osaka, Japan). The reactions, in a total volume of 15 µL, consisted of 7.5 µL of Mastermix, 1 mM each primer, and 2 µL of the diluted cDNA product. After 2 min at 50°C, the DNA polymerase was activated at 95°C for 10 min, followed by 40 cycles at 95°C for 15 s and 60°C for 1 min. The standard curves for RTA and β-actin cDNAs were generated with serial dilutions, and melting curve analysis was used to verify the specificity of the PCR products. The values for the relative quantification were calculated with the ΔΔCt method. The data were normalized to human β-actin levels to compensate for unequal amounts of cDNA in the samples, and are reported as the increase in mRNA accumulation compared to the mock-cell mRNA levels. All reactions were performed three times with a 7900HT Sequence Detection System (Applied Biosystems). As a negative control, each plate contained a minimum of four wells that lacked the cDNA template.

### PCR analysis of progeny virus

To determine whether miRNAs inhibit KSHV replication and reduce the production of progeny viral particles, 293/Bac36 cells were plated in six wells and after their attachment to the dish, the cells were synchronized to G1 phase by culture in serum-free DMEM for 24 h. Serum was then added to a final concentration of 10%. After 12 h, the cells were transfected with miRNAs or their corresponding vectors using Lipofectamine 2000. Four hours after transfection, the medium was refreshed with complete DMEM containing 25 ng/mL phorbol ester and 3 mM valproic acid [29]. The 293/Bac36 cells were incubated for a further five days at 37°C under 5% CO_2_. The supernatants were then collected and passed through a 0.45 µm filter. The viral particles were spun down at 15,000 g for 3 h. The pellet was resuspended in 50 µL of 0.2× PBS, heated to 95°C for 15 min, and then at 56°C for 1 h with protease K (10 mg/mL). The enzyme was then inactivated by heating at 95°C for 30 min. A 5 µL aliquot of the viral lysate was used for the PCR amplification of the KSHV-specific region of ORFK9. The primer pair for the real-time amplification of ORFK9 was: ORFK9-F, 5′-GTCTCTGCGCCATTCAAAAC-3′; ORFK9-R, 5′-CCGGACACGACAACTAAGAA-3′.

## Supporting Information

Figure S1 **Bulge-loop quantitative PCR confirms mature KSHV miRNA expression by miRNA expression constructs.** (A) PCR products were separated on 1.5% agarose gels. Lane 1, 293/Bac36 cDNA; lane 2, cDNA from HEK293 transfected with miRNAs expression constructs; lane 3, HEK293 cDNA; lane 4, H_2_O. Molecular standards are DNA marker DL 2000 (Takara). (B) Relative expression level for all mature miRNAs expressed by constructs compared with miRNAs expressed in 293/Bac36 cells. The expression level of the lowest expressed miR-k12-9 was set as 1 for the controls, and the expression level of other miRNAs was calculated in comparison with miR-K12-9. Data shown are from three independent experiments.
(TIF)

Figure S2 **Relative expression level of miR-K12-7-5p and miR-K12-7-3p in 293/Bac36 cells and 293/Bac36 cells transiently transfected with miR-K12-7 expression construct.** (A) PCR products were separated on 1.5% agarose gels. Lane 1, cDNA from 293/Bac36 transfected with miR-K12-7 expression construct; lane 2, HEK293 cDNA; lane 3, 293/Bac36 cDNA; lane 4, BCBL-1 cDNA; lane 5, H_2_O. (B) Relative expression levels for miR-K12-7-5p and miR-K12-7-3p expressed in 293/Bac36 cells and 293/Bac36 cells transfected with miR-K12-7 expression construct. The expression level of the lowest expressed miR-k12-7-5p in 293/Bac36 cells was set as 1, and expression level in other cells was calculated in comparison with miR-K12-7-5p expressed in 293/Bac36 cells. Data shown are from three independent experiments.
(TIF)

Figure S3 **Raw western blotting data from three independent experiments for** **Figure 3A and 3B** **.**
(TIF)
