# Supplementary figures and images for: miR-K12-7-5p Encoded by Kaposi's Sarcoma-Associated Herpesvirus Stabilizes the Latent State by Targeting Viral ORF50/RTA
Source: PLoS One. 2011 Jan 20;6(1):e16224. doi: 10.1371/journal.pone.0016224 (PMC3024408; doi:10.1371/journal.pone.0016224)

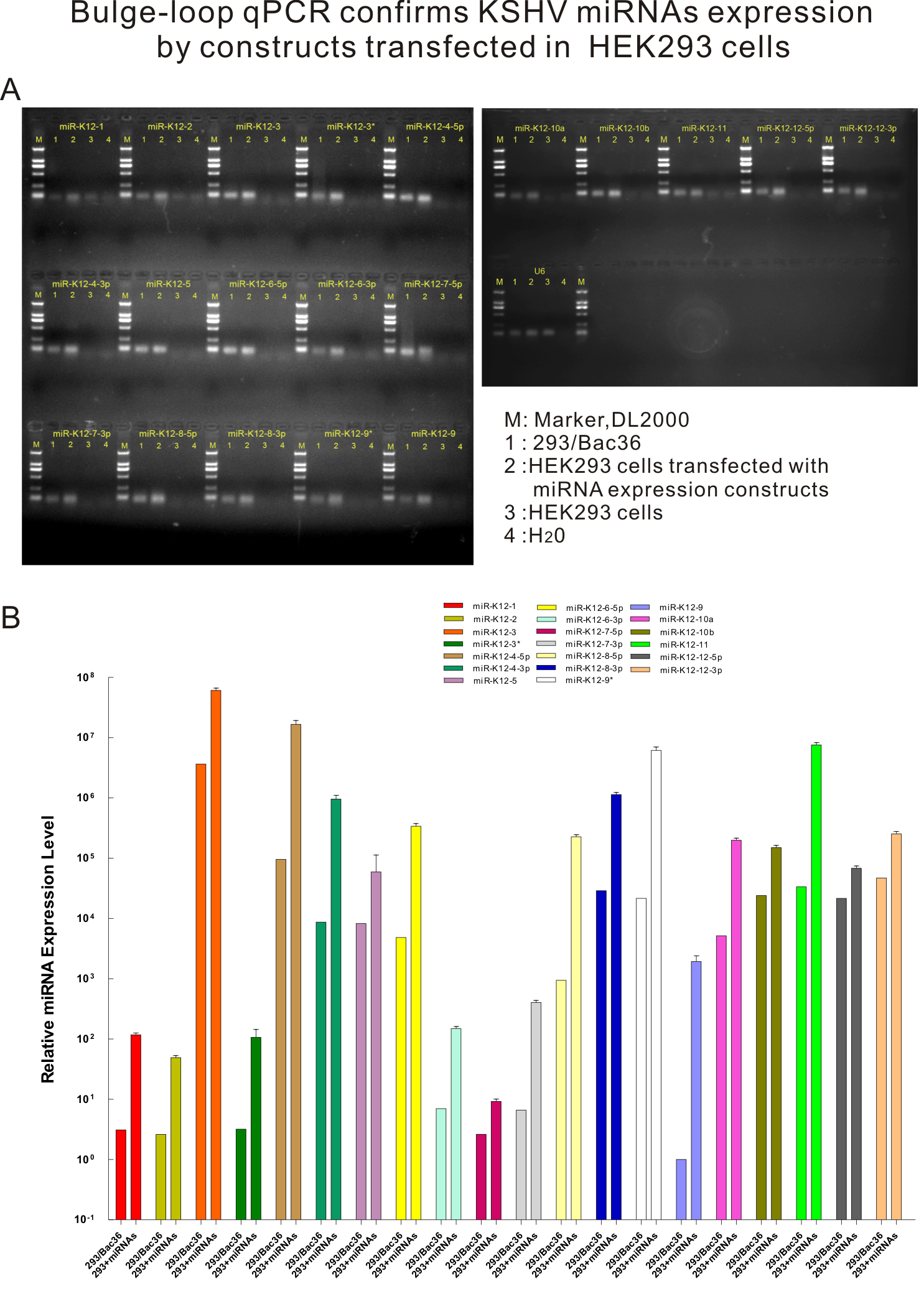

Supplement: Figure S1 — Bulge-loop quantitative PCR confirms mature KSHV miRNA expression by miRNA expression constructs. (A) PCR products were separated on 1.5% agarose gels. Lane 1, 293/Bac36 cDNA; lane 2, cDNA from HEK293 transfected with miRNAs expression constructs; lane 3, HEK293 cDNA; lane 4, H2O. Molecular standards are DNA marker DL 2000 (Takara). (B) Relative expression level for all mature miRNAs expressed by constructs compared with miRNAs expressed in 293/Bac36 cells. The expression level of the lowest expressed miR-k12-9 was set as 1 for the controls, and the expression level of other miRNAs was calculated in comparison with miR-K12-9. Data shown are from three independent experiments. (TIF) [file pone.0016224.s001.tif]

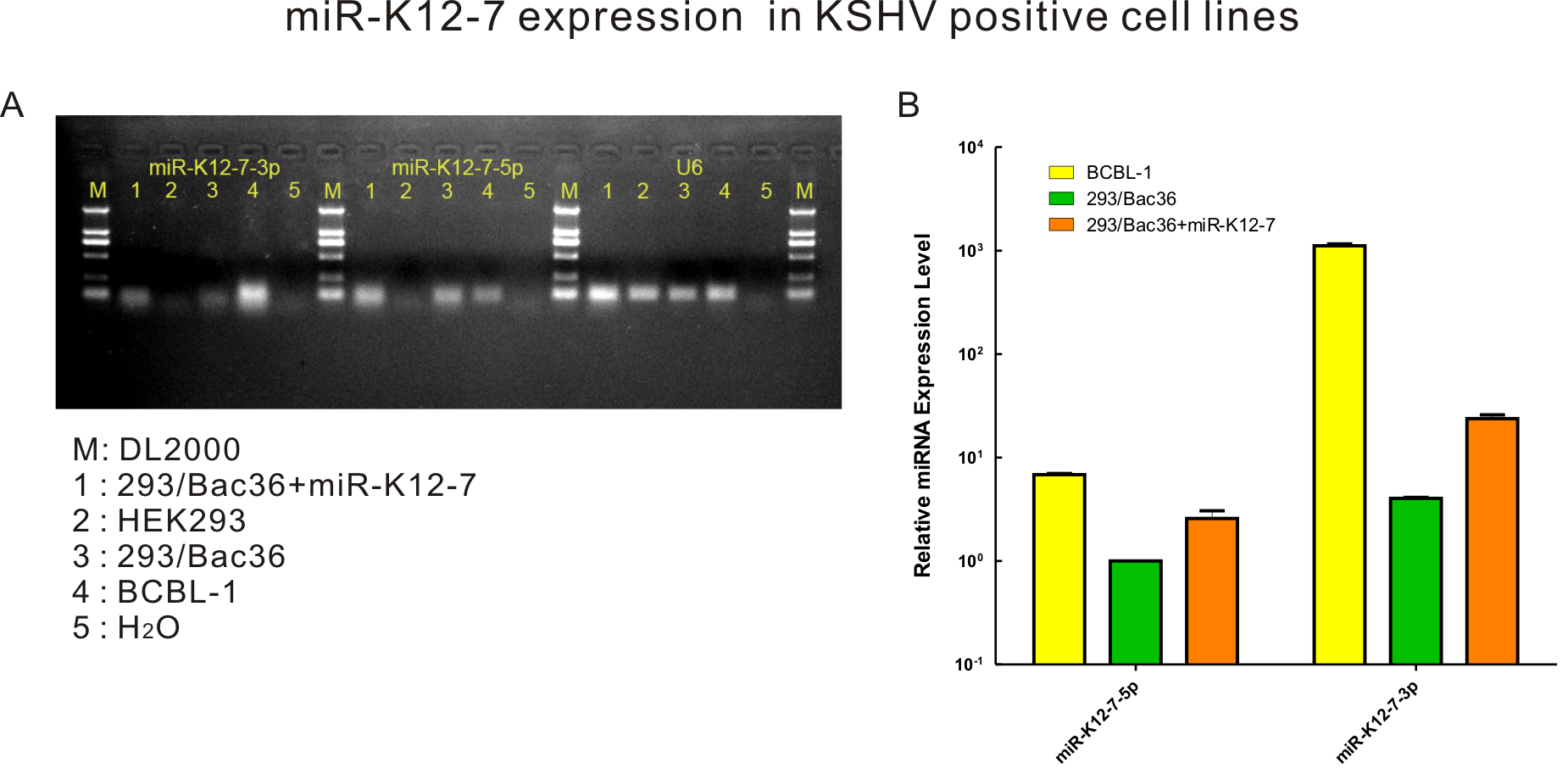

Supplement: Figure S2 — Relative expression level of miR-K12-7-5p and miR-K12-7-3p in 293/Bac36 cells and 293/Bac36 cells transiently transfected with miR-K12-7 expression construct. (A) PCR products were separated on 1.5% agarose gels. Lane 1, cDNA from 293/Bac36 transfected with miR-K12-7 expression construct; lane 2, HEK293 cDNA; lane 3, 293/Bac36 cDNA; lane 4, BCBL-1 cDNA; lane 5, H2O. (B) Relative expression levels for miR-K12-7-5p and miR-K12-7-3p expressed in 293/Bac36 cells and 293/Bac36 cells transfected with miR-K12-7 expression construct. The expression level of the lowest expressed miR-k12-7-5p in 293/Bac36 cells was set as 1, and expression level in other cells was calculated in comparison with miR-K12-7-5p expressed in 293/Bac36 cells. Data shown are from three independent experiments. (TIF) [file pone.0016224.s002.tif]

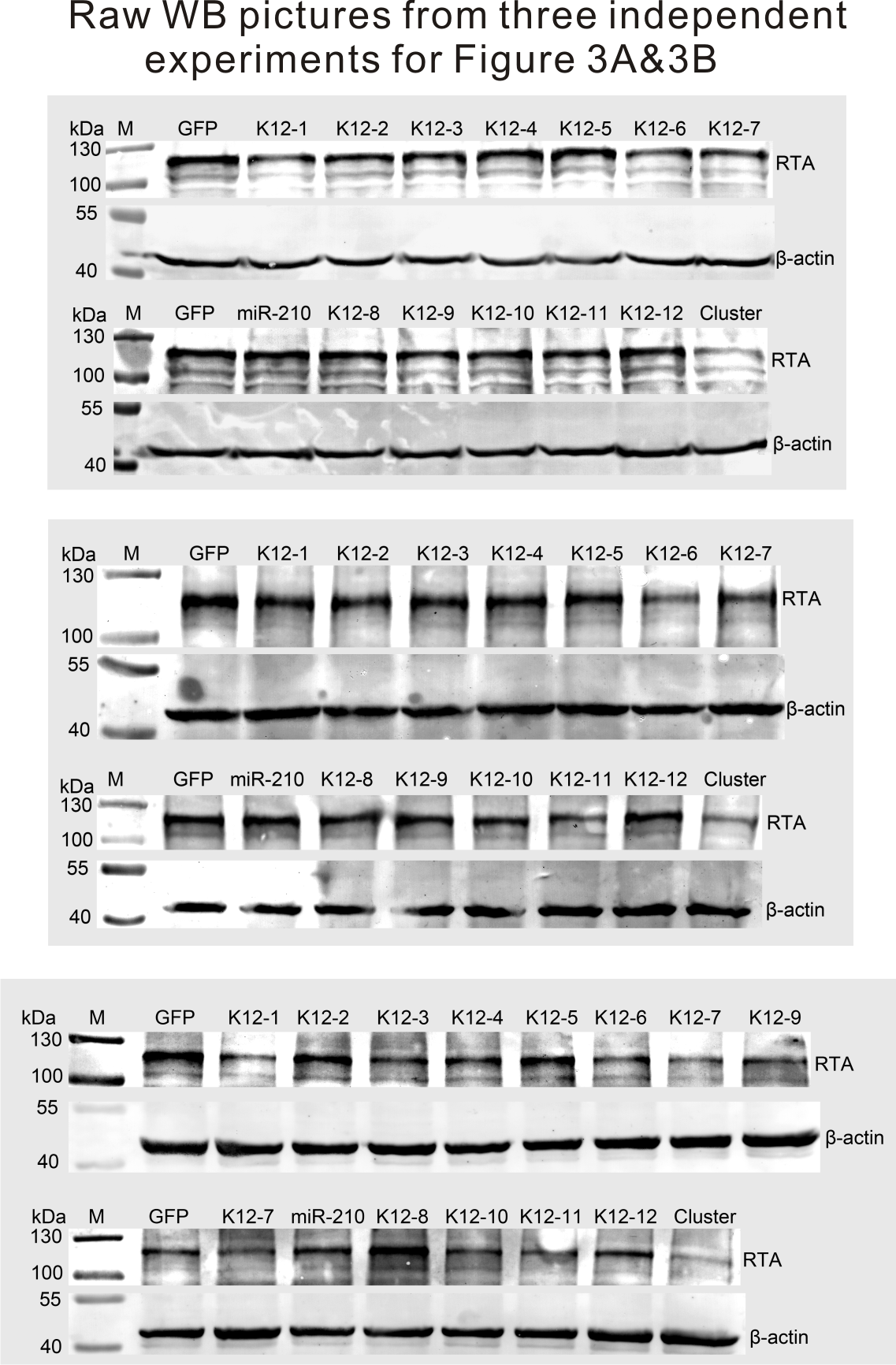

Supplement: Figure S3 — Raw western blotting data from three independent experiments for Figure 3A and 3B . (TIF) [file pone.0016224.s003.tif]
